# Do pregnant women want to know the sex of the expected child at routine ultrasound and are they interested in sex selection?

**DOI:** 10.1080/03009734.2017.1408723

**Published:** 2018-01-04

**Authors:** Margareta Larsson, Minna Berglund, Emelie Jarl, Tanja Tydén

**Affiliations:** Department of Women’s and Children’s Health, Uppsala University, Sweden

**Keywords:** Parents, pregnancy, sex determination, sex selection, ultrasound

## Abstract

**Background:**

The aim of the study was to investigate if expecting parents wanted to know the sex of the fetus during ultrasound examination and if they had discussed it with the midwife. Another aim was to explore any interest in sex selection.

**Methods:**

A longitudinal survey in early and late pregnancy among 2393 women in Sweden.

**Results:**

Almost all (95.8%, *n* = 2289) women had discussed sex determination with the partner before the ultrasound scan, and 57% (*n* = 1356) of women and their partners wanted to find out the fetal sex. The expecting parents mostly initiated a discussion with the midwife (46%, *n* = 1088), but 10% (*n* = 229) stated that the midwives initiated the discussion. Few (5%, *n* = 118) expressed a potential interest in selecting sex of a baby. Women who were interested in sex determination did not differ from those who were not, with respect to age, origin, education, parity, level of pregnancy planning, or importance of religion, but women who had chosen another fetal diagnostic method were more interested in sex determination and in potential sex selection.

**Conclusions:**

Half of women and their partners wanted to know the fetal sex, and 5% were interested in sex selection. This high interest in sex determination is a challenge, since present national guidelines do not include sex determination as an option.

## Introduction

Medical reasons for an ultrasound scan during early pregnancy are to estimate the date of delivery, diagnose multiple pregnancies, detect aberrations in fetal anatomy, identify the location of the placenta, and measure the quantity of amniotic fluid (1). With more sophisticated ultrasound techniques it has become possible to identify fetal sex during an ultrasound scan with high accuracy (2,3). Clients’ reasons have been shown to be somewhat different—to confirm fetal viability, to become informed of any fetal abnormalities, and an opportunity to get to know the child’s sex (4–6). In Sweden all pregnant women are offered a routine ultrasound scan in pregnancy week 15–22 that is free of charge, and almost all (97%) attend this examination (7); 80% of these ultrasound scans are carried out by registered nurse-midwives who are specially trained in the obstetric ultrasound technology.

Internationally, many parents wish to know the sex of the expected child (8–13). According to Shipp et al. (8), 58% of pregnant women in a US study desired to find out the sex prenatally, while studies from Nigeria show that over 90% wanted to know the sex (11,12). There are several reasons for this wish: to plan for the baby’s arrival, acquire appropriate things for the child, personal and partner’s curiosity, and to make sure sex determination at birth is correct (12,14,15).

Few studies from the Nordic countries have investigated prenatal sex determination or sex selection. However, in a Swedish study the majority of pregnant women considered it unimportant to know the sex, yet 57% of study participants chose to find out (16). In another study (17), 13% of women and 17% of men believed that a reason to do an ultrasound was to reveal the sex of the fetus. According to the National Board of Health and Welfare, prenatal testing for sex determination should not be offered without medical indication, and the sex of a fetus should be disclosed only if the woman requests it (18).

Prenatal attachment is a theory about the relationship between expecting parents and their unborn children, and the parents’ behavior in relation to the fetus (19). Prenatal attachment can be divided into dimensions that differ depending on the trimester of the pregnancy. Prenatal attachment plays a major role during pregnancy, and in the postnatal period it affects the mothers’ ability to give good child care (20). The ultrasound examination may contribute to the prenatal attachment (20–22). One study shows that health-care professionals consider sex determination as a key part of the attachment to the fetus (23).

Factors associated with the desire to know the sex of the baby were: unplanned pregnancy, previous experience of knowing the sex of an expected child, not planning to breastfeed, low socioeconomic status, being unmarried (9,10), being younger than 22 or older than 40 years (8,9), and considering that the sex of the baby affects family planning. Women who had a perfectionist view of parenting were more likely to have found out the baby’s sex (10). It was more common to have prenatal sex determination among women with prior deliveries compared to first-time mothers (8,24), and those who previously had a boy and a girl more often chose to find out the sex (8). Shipp et al. (8) showed that couples who had chosen prenatal sex determination agreed on the decision.

Sex determination in early pregnancy has been feared to be a risk for selective abortion (25), especially in countries with a son-preference such as China and India (24,26). People with low education, low socioeconomic status, and women who already have children are more likely to prefer a certain sex of the expected child. In Western countries preference for boys or girls is not as clear, and sex selection would probably not affect the ratio between the percentage of girls and boys (27). In the US, the UK, and Germany, people have more negative attitudes toward sex selection compared to China and India (28).

No recent Swedish study has investigated the wish to find out the sex of the fetus at the routine ultrasound scan. The main aim of the present study was to investigate if pregnant women and their partners wanted to know the sex of the expected child, if this had been discussed with the midwife during the examination, and if there were any differences related to age, education, having previous children, planned/unplanned pregnancy, and importance of religion. Another aim was also to explore any interest in sex selection. We hypothesized that women with a very planned pregnancy would be more interested in knowing the sex of the fetus compared to women with less planned pregnancies.

## Method

This cross-sectional study is based on selected items from a Swedish longitudinal study on health and lifestyle before, during, and after pregnancy—The Swedish Pregnancy Planning Study (SWEPP). The study procedure has been described by Stern et al. (29) and Bodin et al. (30). A total of 215 antenatal clinics were invited to participate in the data collection. Of these, 71% (*n* = 153) accepted, and recruitment took place between September 2012 and July 2013.

Participants gave their written consent, and the midwives kept logs over the enrollment process. Women answered three questionnaires; the first (Q1) in early pregnancy, the second (Q2) in late pregnancy, and the third (Q3) one year post partum. Variables regarding background and level of pregnancy planning were retrieved from Q1, and items regarding the desire to find out the fetal sex were collected in late pregnancy (Q2).

In total 5796 women visited the clinics during the study period. Of these, 303 were not invited to participate in the study for various practical reasons. Thus, 5493 women were invited, and 62% (*n* = 3389) participated. We sent a postal questionnaire (Q2) to 3215 women who had responded to Q1 and had revealed their contact details, and of these 77% (*n* = 2583) returned Q2. An analysis of those who dropped out showed no differences with respect to age, education, religion, or previous children. In total, 2405 of the women responded that they were still pregnant, and, among those, 2393 had attended the routine ultrasound scan in early pregnancy. These women thus formed the sample for the present study.

The questionnaire consisted of 148 questions, most of which were multiple choice questions. The questionnaire was designed by researchers and clinicians, and some items were adjusted after a pilot study (31).

Questions regarding the demographic background used in this study covered the woman’s age, origin, highest completed education, monthly income, number of previous children, and the importance of religion. Pregnancy planning in this study refers to whether the pregnancy was planned before conception and was measured with the 5-grade Swedish Pregnancy Planning Scale (SPPS): very planned, fairly planned, neither planned nor unplanned, fairly unplanned, and very unplanned. This SPPS has been used in previous studies (29,30,32).

Items presented and analyzed in the present paper were phrased as follows:

- From where did you and/or partner look for information about pregnancy? (Eleven pre-defined options/One open option/Have not looked for any information)
- Before the ultrasound examination, did you and your partner discuss if you wanted to know the child’s sex? (Yes/No)
- Did you want to know the child’s sex? (Yes/No)
- Did your partner want to know the child’s sex? (Yes/No)
- Did you and the midwife discuss the opportunity to identify the child’s sex? (Yes, on my/my partner’s initiative/Yes on the midwife’s initiative/No/Do not remember)
- Do you know the child’s sex today? (Yes, a boy/Yes, a girl/No, did not want to know/No, was not allowed to know)
- If it was possible, would you take advantage of the possibility to select the sex of your baby? (Yes/No/Do not know)
- Did you go through any other fetal diagnostic method in addition to the routine ultrasound? (Yes/No)

The regional ethical review board in Uppsala, Sweden, approved the study (Dnr: 2010/085).

### Data analysis

The participants were divided into three age-groups: ≤24 years, 25–34 years, and ≥35 years. The variable regarding importance of religion had five response alternatives, which were collapsed into three: great importance/indifferent/little importance. The highest completed education was collapsed into low/high education, and the monthly income was collapsed into low/high income.

To analyze differences between those who wanted to find out the sex of the fetus and those who did not, and between those potentially interested in sex selection and those who were not, we used the chi-square test and Fisher’s exact test for nominally and ordinally scaled variables. The *t* test was used to analyze any age difference. A *p* value of *p* < 0.05 was chosen.

## Results

A majority of the participants belonged to the age-group 25–34 years (mean age 29.4 years), and more than 90% had a Nordic origin. Almost half (48%) had a university education, and 54.7% already had one or more children. Most pregnancies (75.5%) were very or fairly planned. A minority (11.5%) stated that religion was of great importance to them (Table 1).

**Table 1.** Characteristics of the respondents (*n* = 2393). Missing values excluded from the analysis.

	*n*	%
Mean age (range), years^a^	29.4 (17**–**47)	
Age-group		
≤24 years	377	16.1
25–34 years	1625	69.4
≥35 years	341	14.6
Origin		
Nordic	2203	92.2
Non-Nordic	186	7.8
Income		
High	1110	47.5
Low	1227	52.5
Previous children		
No previous children	1085	45.3
Has previous children	1308	54.7
Gone through another method of fetal diagnostics	984	41.1
Education		
Primary school (9 years)	128	5.4
Secondary school (12 years)	927	39
Vocational education	181	7.6
University <2.5 years	108	4.5
University >2.5 years	1035	43.5
Pregnancy planning		
Very planned	1126	47.5
Fairly planned	665	28.0
Netiher planned nor unplanned	319	13.4
Fairly unplanned	77	3.2
Very unplanned	186	7.8
Importance of religion		
Great importance	273	11.5
Indifferent	530	22.3
Little importance	1575	66.2

a Age range: Minimum age of respondents was 17 years and maximum age was 47 years.

Most women had searched for information in early pregnancy, and the most cited sources of information were different Internet fora, followed by the antenatal clinic (Table 2). Before the routine ultrasound scan 95.8% (*n* = 2289) of the women had discussed with their partner whether they wanted to know the sex of the fetus, and an equal number of women (56.9%, *n* = 1356) and partners (56.9%, *n* = 1359) wanted to find out their baby’s sex. After the ultrasound scan, 48.1% (*n* = 1152) responded that they did not know the sex of the expected child (Table 3).

**Table 2.** Sources of information used by the participants in early pregnancy (*n* = 2393). Missing values excluded from the analysis.

Sources of information^a^	*n* (%)
Internet fora targeting expectant parents	967 (40.4)
Internet site for health-care information	679 (28.4)
Antenatal clinic	613 (25.6)
Family and friends	510 (21.3)
The National Food Agency	416 (17.4)
Mobile applications	466 (19.5)
Other Internet sources	316 (13.2)
Books	244 (10.2)
Newspapers	223 (9.3)
Blogs	176 (7.4)
Other health services	66 (2.8)
National Public Health Agency	18 (0.8)
Did not look for any information	846 (35.4)

a Several alternatives could be chosen.

**Table 3.** Interest in sex determination and sex selection (*n* = 2393). Missing values excluded from the analysis.

Item	*n* (%)
Discussed if they wanted to know the sex of the baby before the ultrasound examination	2289 (95.8)
Pregnant woman wanted to know the sex of the baby	1356 (56.9)
Partner wanted to know the sex of the baby	1359 (56.9)
Discussed sex determination with the midwife	
** **On own/partner’s initiative	1088 (45.5)
** **On the midwife’s initiative	229 (9.6)
Knows the sex of the baby	1241 (51.9)
Interested in sex selection	118 (4.9)

During the ultrasound scan 45.5% (*n* = 1088) of the couples and 9.6% (*n* = 229) of the midwives initiated a discussion about sex determination. Four out of 10 (39.5%, *n* = 943) did not discuss this, and 5.4% (*n* = 130) did not remember whether or not the possibility of sex determination was discussed. Most women (84%, *n* = 2007) responded that they would not use the possibility to select the sex of a baby if it was possible; 5% expressed such an interest, and 11% were unsure.

Women who wanted to find out the sex of the baby did not differ from those who did not, in any of the analyzed characteristics—age, origin, highest completed education, monthly income, previous children, importance of religion, or level of pregnancy planning (Table 4). Neither did women potentially interested in sex selection differ from women who were not, in any of the above-mentioned variables (data not shown). However, women who also had gone through another method of fetal diagnostics were more interested in finding out the sex of the fetus (*p* < 0.01) and in a potential possibility to select the sex of a child (6.2% versus 4.1%, *p* < 0.01).

**Table 4.** Comparisons between women who wanted to know the sex of the baby versus those who did not (*n* = 2393). Missing values excluded from the analysis.

Item	Wanted to know the sex of the baby	Did not want to know the sex of the baby	*p* value
Mean age, years	29.4	29.4	0.80
	*n* (%)	*n* (%)	
Age groups			0.99
** **17–24 years	214 (16.1)	160 (15.9)	
** **25–34 years	923 (69.4)	697 (69.4)	
** **35–47 years	193 (14.5)	148 (14.6)	
Origin			0.45
** **Nordic	1245 (92.1)	950 (92.3)	
** **Non-Nordic	107 (7.9)	79 (7.7)	
Education			0.32
** **Low	584 (43.3)	465 (45.5)	
** **High	764 (56.7)	558 (54.5)	
Previous children			0.43
** **No previous childen	624 (46.0)	456 (44.3)	
** **Has previous children	732 (54.0)	573 (55.7)	
Income			0.53
** **Low	620 (46.9)	485 (48.2)	
** **High	703 (53.1)	521 (51.8)	
Pregnancy planning			0.34
** **Very/fairly planned	1004 (74.7)	781 (76.4)	
** **Neither planned nor unplanned	180 (13.4)	139 (13.6)	
** **Very/fairly unplanned	160 (11.9)	102 (10.0)	
Gone through another method of fetal diagnostics	594 (43.8)	388 (37.7)	<0.001
Importance of religion			0.44
** **Great importance	146 (10.9)	127 (12.4)	
** **Neither important nor unimportant	296 (22.0)	231 (22.5)	
** **Little importance	903 (67.1)	667 (65.1)	

## Discussion

An interesting finding was that almost all women (96%) had discussed with their partner before attending the ultrasound scan whether they wanted to find out the fetal sex, and slightly more than half of the women (57%) and a similar proportion of their partners desired to find out the fetal sex. In almost half of the cases it was the couple who brought up the possibility of sex determination with the midwife. Equally interesting in an international perspective is the fact that almost half of the respondents had not, or did not remember if they had, discussed this issue during the ultrasound examination.

The proportion of women who wanted to find out the fetal sex during routine ultrasound is in line with Sjögren (16). This suggests that the desire to know the sex in Sweden is as common today as it was 29 years ago, even though the ultrasound technique has developed over the years and private clinics offering this service are available at least in some cities. The proportion of women who wished to know the child’s sex is also consistent with what Shipp et al. (8) showed in the study from the US. It was, however, less common compared to the studies from Nigeria (11,12). This may be due to a greater similarity between Sweden and the US compared with Nigeria. The sampling procedure may also have been different.

The proportion of partners who wanted to know the child’s sex during routine ultrasound was the same as the proportion of the pregnant women, which indicates a common decision-making, which also a previous study noted (8). Almost all had discussed with their partner about sex determination before the routine ultrasound scan. This demonstrates that expecting parents in Sweden are aware of the possibility to identify the sex of the expected child during the ultrasound scan. It also indicates that a pregnancy is regarded as a joint project, which was also shown by Bodin and co-workers (30,33).

Most discussions about sex determination were initiated by the couple, but in some cases the midwife raised the issue. This is not in accordance with national guidelines, but these midwives may see the ultrasound scan as an important part of prenatal attachment, as demonstrated in a previous study (23). However, from the midwife’s perspective, the intention to identify the sex during a routine ultrasound is secondary to the medical examination. Determining the sex is sometimes difficult, depending on the weight of the woman and the position of the fetus, and the examination may therefore demand more time (34). Many parents want to know the sex, but the caregiver is only allowed to disclose the sex of the expected child if the pregnant woman expresses such a wish. Recall bias, regarding who initiated the discussion, cannot be ruled out, since the women responded to the question in the third trimester and the actual examination took place in the second trimester.

In our study there was no difference in the desire to know the baby’s sex with respect to age. This is in contrast to previous studies showing that age is important for prenatal sex determination (8). The importance of religion had also no bearing on the desire to know the baby’s sex, but our question about the importance of religion was not linked to a specific religion. According to Shipp et al. (8) Catholics were less inclined to find out the baby’s sex compared to people belonging to another religion. Neither did the level of education influence the desire to know the sex, which is in contrast to previous studies showing that low education was associated with higher levels of prenatal sex determination (8,10,24).

In the present study there was no difference in the level of pregnancy planning between those who wanted to know the sex of the baby and those who did not. This is in contrast to other studies showing that women with an unplanned pregnancy were more keen to find out the sex of the expected child (9,10). We can only speculate about the reason for this difference. One possible explanation is that the proportion of unplanned pregnancy in our sample was low, and even though some pregnancies were unplanned they were probably not unwanted since the woman/couple had decided to carry the pregnancy to term.

In addition, we could not demonstrate any difference regarding previous children or not between those who wanted to know the sex of the baby and those who did not. In the study from India more women with prior deliveries wished to find out the baby’s sex, compared with first-time mothers (24). In India sex determination is illegal. Nonetheless, most pregnant women know the sex of their baby, and it has been estimated that around seven million girls are aborted every year (35). In Western countries, there is not such an out-spoken preference for male offspring (27), which can explain that there was no difference between first-time pregnant women and those who already had children. However, an American study also showed that it was more common among women with prior deliveries to find out the sex prenatally, especially if they only had one previous child (8).

The finding that women who had gone through another method of fetal diagnostics were more interested both in sex determination and sex selection did not come as a surprise. In a subsample of this cohort and their partners, an association between an interest in preconception genetic carrier screening (PCS), experiences of prenatal diagnostics, and wanting to find out or select the sex of their child was found (36).

This study focused on the desire to find out the sex during a routine ultrasound scan. Another non-invasive way to perform prenatal sex determination is by non-invasive prenatal diagnosis (NIPD). This means that fetal DNA is analyzed in a maternal blood sample, and the test can be performed already in pregnancy week seven. The method is not yet used for sex determination of the fetus in Sweden, but it has created an ethical discussion on prenatal sex determination. This new technique is expected to lead to increased sex selection in developed countries where this test is available (25). In Sweden, any woman can choose to terminate a pregnancy until the end of the eighteenth week of gestation without having to disclose any reason for the abortion. According to the National Board of Health (37) 94% of all abortions in 2016 in Sweden were performed before 12 weeks, and 53% before pregnancy week seven. These high numbers of very early induced abortions indicate that an abortion decision is taken very early in pregnancy and in most cases because the pregnancy is unwanted or mistimed (38). More studies on the effects of early sex determination in Sweden are needed to rule out the potential risk that abortions can be carried out because of the sex of the fetus.

Wanting to know their child’s sex is not the same as having a preference for a particular sex. However, according to Shipp et al. (8), prenatal sex determination is much more common among those who have a preference for one sex compared with those who have no such preference. In our study, very few wanted to be able to decide on the sex of a future child. However, prenatal sex determination is a prerequisite for sex selection (39). This reluctance toward sex selection is in line with the study about potential interest in PCS, showing that both women and men had relatively high uncertainty toward PCS (35). Around half of the women were opposed to such selection of a child, and they were also concerned about negative consequences. Other ethical aspects, such as justice and autonomy, have also been raised in relation to PCS (40). The expanding use of fetal diagnostics technology worldwide calls for increased access to high-quality counseling already before pregnancy—so-called preconception care (41). Such counseling can provide future parents with unbiased information so they can make well-informed decisions once a pregnancy has occurred.

### Strengths and limitations

The strength of the study is the high number of participants and the broad recruitment from many different ANCs in the country. Since almost 100% of pregnant women attend ANCs in Sweden, our sample is most probably representative for the entire Swedish-speaking population. However, we have no information about women who were not invited to participate or declined participation. Another limitation was that only the pregnant women responded, also on behalf of their partners’ wishes.

We have no information about the sex of the women’s previous children so we could not analyze the wish for sex determination against the background of the sex of previous children. Only Swedish-speaking women could respond to Q2, which limits the possibility to generalize the results to all women living in Sweden.

## Conclusions

More than half of all pregnant women and their partners wanted to find out the sex of the expected child during the routine ultrasound scan. This percentage is low from an international perspective, but is still a challenge, since present national guidelines do not include sex determination as an option. We need more national and international studies on sex determination during pregnancy for further knowledge of how the desire to find out the sex of the baby is shaped and what this development might lead to in the future.
